# Beyond Entropy: Organization as the Preservation of Structural Identity

**DOI:** 10.3390/e28080876

**Published:** 2026-08-04

**Authors:** Ricardo J. Silva

**Affiliations:** 1 Department of Computing Sciences, Villanova University, Villanova, PA 19085, USA; ricardo.silva@villanova.edu; Tel.: +1-321-337-1120; 2 Foundation for Living, Wellness, and Health, 2093 Philadelphia Pike #1065, Claymont, DE 19703, USA

**Keywords:** organization, structural identity, organizational classes, entropy, recognition boundary, generalized state space, communication systems, observability, organizational deviation

## Abstract

This work introduces a framework in which organization is defined as the degree to which identity-defining relationships among system states are preserved under transformation or perturbation. Existing descriptors such as energy, entropy, mutual information, and divergence measures characterize magnitude, statistical dispersion, dependency, and deviation, yet do not explicitly address the persistence of recognizable structure. To address this limitation, the concepts of organizational classes, reference organizational models, organizational deviation, and recognition boundaries are introduced within a generalized state-space representation. The proposed framework treats recognizable structures as members of organizational classes whose identities are determined by defining constraints and relationships rather than by specific physical realizations. A probabilistic implementation is developed in which organizational classes are represented by reference models and organizational preservation is estimated through measures of organizational deviation. Recognition is incorporated through observer-dependent recognition boundaries that determine whether a realization remains identifiable as a member of a given class. The framework is illustrated through geometric, perceptual, and communication-based examples, including structural degradation in a maximum-entropy background, observer-dependent recognition, channel-limited observability, and a quantitative Gaussian organizational model. These examples demonstrate that entropy and organization are complementary descriptors that may evolve independently: organizational identity may degrade while occupancy statistics remain largely unchanged. The results suggest that communication and sensing systems may be interpreted not only as processes that transport energy or information, but also as systems that preserve, transform, or degrade organizational structure. By providing a descriptor for structural identity alongside entropy and information, the proposed framework offers a foundation for studying how organized structures emerge, persist, transform, and degrade across a wide range of physical, informational, and complex systems.

## 1. Introduction

Signal processing and communication systems are fundamentally concerned with the transmission, transformation, storage, and interpretation of signals in the presence of noise, distortion, and physical constraints. Classical formulations describe signals using temporal, spectral, spatial, and phase-space representations, while system behavior is characterized through quantities such as energy, power, bandwidth, signal-to-noise ratio (SNR), entropy, mutual information, and channel capacity [1,2,3,4,5,6,7,8,9,10]. Together with electromagnetic field theory and stochastic process models, these concepts form the foundation of modern communication, sensing, imaging, and information-processing systems [1,2,3,4,5,6,7,8,9,10].

Within this framework, energy and power quantify signal magnitude, entropy characterizes statistical dispersion and uncertainty, and information-theoretic measures quantify the ability of systems to transmit or infer information [3,9,10]. Advances in coding, modulation, coherent communications, and signal processing have enabled modern systems to approach theoretical performance limits in increasingly complex environments [11,12,13,14]. More recently, semantic communication systems have highlighted the growing importance of structure and contextual relationships in communication tasks, extending the discussion beyond purely symbol-level fidelity [15].

At the same time, active research continues to explore new perspectives on information, probability distributions, invariant measures, and entropy itself. Recent contributions have examined improved estimators of mutual information, alternative representations of probability distributions, invariant probability measures in stochastic systems, and broader classifications of entropy across scientific disciplines [16,17,18,19]. These developments reflect a continuing effort to develop richer descriptive frameworks capable of capturing increasingly complex forms of system behavior.

A recurring theme across many disciplines is the recognition that system behavior cannot always be understood solely through the properties of individual components. Complex systems frequently exhibit hierarchical structure, where higher-level phenomena emerge from relationships among simpler elements rather than from the elements themselves. Simon’s seminal work on the architecture of complexity emphasized the importance of hierarchical organization and nearly decomposable structures in understanding complex systems across physical, biological, and social domains [20,21]. Under this perspective, meaningful system descriptions often arise not from individual states alone, but from the relationships that connect them.

Similar ideas have emerged in modern representation theory and machine learning. Geometric deep learning, equivariant neural networks, and invariant representation learning are founded on the observation that successful representations frequently depend upon preserving symmetries, invariances, and structural relationships under transformation [22,23,24]. In many recognition tasks, the identity of an object is preserved despite changes in scale, orientation, position, appearance, or representation. Such observations suggest that recognizable structures may be characterized not merely by the states they occupy, but by the relationships that remain preserved across transformations.

The same principle appears in studies of emergence and complex systems, where higher-level properties arise from correlations among interacting components [25]. A collection of isolated elements may possess little significance individually, yet exhibit recognizable organization when appropriate relationships are established among them. In such systems, identity often emerges as a collective property that cannot be attributed solely to any individual component. This observation suggests that the preservation of relationships may be as important as the preservation of states themselves.

Despite these developments, existing descriptors remain primarily focused on quantities such as magnitude, uncertainty, dispersion, statistical dependence, or transport between probability distributions. Energy characterizes the magnitude of activity, entropy measures occupancy dispersion, divergence measures quantify differences between distributions, and mutual information measures statistical dependence [3,9,10,16,17,18,19,26,27,28]. While these descriptors have proven extraordinarily successful, none explicitly quantify the persistence of recognizable structure under transformation or perturbation.

This limitation becomes particularly evident in systems whose functionality depends on preserving structural identity. Interference, resonance, coherent communications, pattern recognition, imaging systems, coding schemes, and semantic communication all rely on the preservation of relationships among system states rather than on energy alone [13,14,15,22,23,24,25]. Two systems may possess similar energy, entropy, or statistical characteristics while exhibiting dramatically different abilities to preserve recognizable structure. Conversely, substantial transformations may occur while preserving the relationships necessary for recognition.

These observations motivate the introduction of an additional descriptive framework concerned explicitly with the persistence of recognizable structure. Rather than focusing exclusively on occupancy, uncertainty, or energetic magnitude, such a framework should provide a means for characterizing how well the defining relationships of a system survive transformation, degradation, or perturbation.

The objective of this work is therefore to develop a probabilistic framework for describing and quantifying the persistence of recognizable structure in generalized state spaces. The proposed formulation builds upon established concepts from signal processing, information theory, statistical inference, and pattern recognition while providing a complementary perspective focused on structural preservation. The theoretical foundations of this framework are introduced in the following section, where the concept of organization is developed as a descriptor of structural identity prior to its mathematical formulation and quantitative evaluation.

## 2. Organization as a Descriptor of Structural Identity

### 2.1. Identity and Organizational Classes

The preceding discussion established that many systems remain recognizable despite undergoing significant transformations. A geometric shape may be rotated or scaled, a communication signal may be amplified or attenuated, a message may be translated or partially corrupted, and a biological structure may experience continuous turnover of its constituent components while retaining its functional identity. In each case, the system remains identifiable despite changes in its specific physical realization.

This observation suggests that identity is not determined solely by individual states but by the persistence of a set of defining relationships that characterize membership within a recognizable class. A five-pointed star, for example, is not defined merely by the existence of vertices and line segments. Rather, it is defined by the relationships among those elements. Preserving the number of vertices while arbitrarily perturbing their arrangement may produce a configuration that no longer resembles a star, despite retaining the same constituent elements.

To illustrate the concepts developed throughout this work, a five-pointed star is adopted as the canonical reference structure. The choice is motivated by its simplicity, familiarity, and clearly identifiable geometric relationships. The star serves as an illustrative organizational class whose identity can be readily evaluated under transformation and perturbation.

Importantly, the framework developed in this paper is not specific to geometric figures. The star is used solely as a convenient visual example. The same principles apply to communication constellations, coded messages, biological structures, symbolic systems, spatial patterns, and other organized configurations. In each case, organization is not determined by the individual components themselves, but by the relationships that define membership within a recognizable class.

We therefore introduce the notion of an organizational class, defined as the collection of all configurations that preserve the relationships necessary to maintain a recognizable identity. Different members of an organizational class may exhibit substantial variation in appearance while remaining identifiable as manifestations of the same underlying structure.

**Figure 1:**
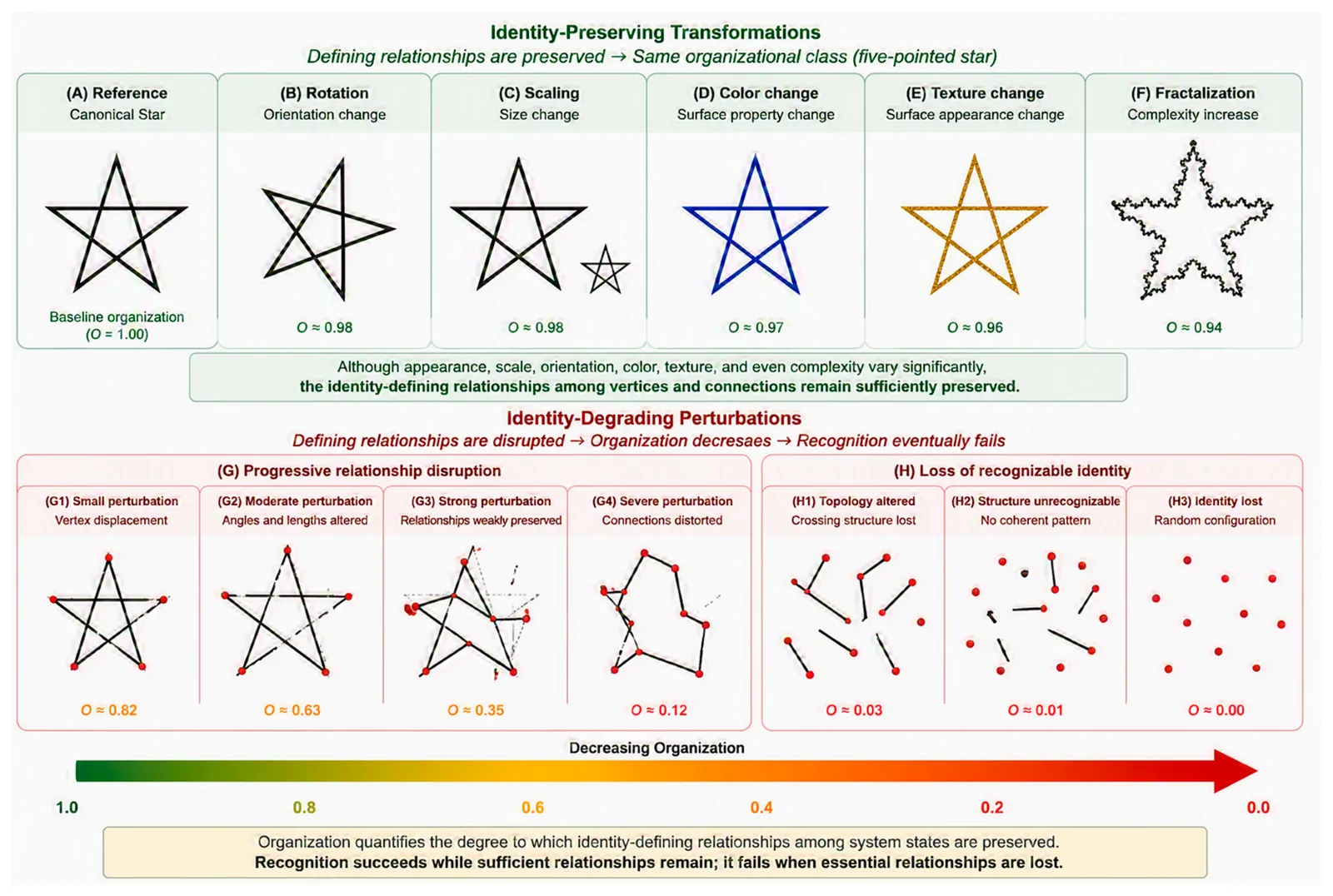
Identity-preserving and identity-degrading transformations of an organized structure. (**A**) Reference five-pointed star defining the organizational class. (**B**–**F**) Identity-preserving transformations including rotation, scaling, color variation, surface modification, and fractal enhancement. Although appearance and complexity change, the defining relationships remain sufficiently preserved for recognition. (**G**,**H**) Identity-degrading perturbations in which the correlations among vertices and connecting segments are progressively disrupted. Despite retaining many of the original elements, the structure eventually ceases to be recognizable as a member of the original organizational class.

Figure 1 illustrates this concept through a sequence of transformations applied to a five-pointed star. Several transformations modify the appearance of the structure while preserving its identity, whereas others alter the defining relationships sufficiently to degrade recognition. The figure highlights the distinction between structural variation and organizational degradation, which constitutes a central principle of the proposed framework.

The figure demonstrates that organization is associated with the preservation of identity-defining relationships rather than the preservation of individual elements, exact geometric coordinates, or specific physical realizations.

### 2.2. Identity-Preserving Transformations

A defining characteristic of organized systems is their ability to preserve identity under transformation. Many transformations alter the appearance of a structure without altering its organizational class. Examples include:

- Translation;
- Rotation;
- Scaling;
- Changes in color;
- Changes in texture;
- Changes in representation;
- Partial degradation.

As shown in Figure 1, a five-pointed star remains recognizable after rotation, scaling, or recoloring because the relationships that define its identity remain substantially unchanged. In contrast, relatively small perturbations to those defining relationships may destroy recognition entirely.

This distinction highlights an important principle: Not all transformations are organizationally equivalent. Some transformations preserve identity, while others alter or destroy it.

The distinction between these two categories forms the foundation of the organizational framework developed in this work. Identity-preserving transformations maintain the defining relationships of an organizational class, whereas identity-degrading transformations disrupt those relationships. Organization is therefore concerned not with change itself, but with the extent to which recognizable identity survives change.

### 2.3. Organizational Constraints

The ability of a structure to preserve identity depends on the constraints that define its organizational class.

These constraints may take many forms, including:

- Geometric constraints, defining spatial relationships among components.
- Symmetry constraints, defining invariant transformations.
- Topological constraints, defining connectivity relationships.
- Probabilistic constraints, defining characteristic occupancy distributions.
- Symbolic constraints, defining grammatical, syntactic, or coding relationships.
- Functional constraints, defining behaviors necessary for system operation.

Different systems may therefore realize organization through different organizational primitives. A crystal lattice, a modulation constellation, a biological regulatory network, a sentence, and a geometric figure all derive their recognizable identities from different sets of constraints.

This perspective provides a common framework for discussing organization across domains. Geometry, topology, symmetry, coding, and functional relationships are not competing descriptions of organization; rather, they represent different mechanisms through which organizational constraints are expressed.

Organized systems may therefore exhibit very different physical realizations while sharing a common characteristic: the preservation of identity-defining relationships under transformation.

### 2.4. Definition of Organization

The preceding discussion motivates a distinction between structure and organization. A structure consists of elements and the relationships among them. However, not all relationships contribute equally to recognizable identity. Some relationships are incidental, while others are essential for preserving membership within an organizational class.

In this work, organization is defined as: The degree to which the identity-defining relationships among system states are preserved under transformation or perturbation.

Under this definition:

- States provide the constituent elements of a system.
- Relationships connect those elements.
- Constraints determine admissible configurations.
- Identity distinguishes membership within a recognizable class.
- Organization quantifies the persistence of that identity.

This interpretation differs from traditional descriptors. Entropy characterizes the dispersion of occupancy probabilities but does not directly describe whether identity-defining relationships remain intact [3,4,10,19]. Mutual information characterizes statistical dependence but does not explicitly determine whether a recognizable structure has been preserved [10,16]. Divergence measures differences between distributions but do not define the identity being preserved [9,26,27,28].

Organization therefore addresses a distinct question: To what extent does a system remain identifiable as a member of a recognizable structural class? This definition is intentionally independent of any specific mathematical formalism. Different organizational systems may require different representations and different measures of deviation. The mathematical framework developed in the following section provides one probabilistic approach for operationalizing this concept.

### 2.5. Organization and Recognition

The preservation of identity becomes meaningful only through recognition. A structure may continue to exist physically while no longer remaining recognizable to an observer, classifier, or communication receiver. Conversely, substantial distortions may occur while sufficient organizational relationships survive to permit successful reconstruction.

Organization should therefore be viewed as an inherently relational quantity. It depends not only on the observed structure but also on the reference framework used to evaluate identity.

This perspective is consistent with established theories of detection, classification, and pattern recognition, where recognition depends on both the observed signal and the capabilities of the observing system [29,30,31]. The same physical structure may therefore yield different recognition outcomes when evaluated by observers possessing different capabilities, prior models, or decision criteria.

Importantly, recognition does not require perfect preservation of structure. Many systems remain identifiable despite substantial degradation because enough identity-defining relationships survive to permit reconstruction. A partially obscured geometric figure, a noisy communication symbol, a damaged code sequence, or a misspelled word may still be recognized when sufficient organizational constraints remain intact.

The organizational framework proposed here is therefore built upon three interconnected concepts:

1. Identity, which defines membership within a recognizable class.
2. Organization, which measures the preservation of identity-defining relationships.
3. Recognition, which determines whether sufficient organizational structure remains to identify the system as belonging to that class.

The following section develops a mathematical framework for representing organizational classes, quantifying organizational degradation, and defining recognition boundaries within generalized state spaces.

## 3. Mathematical Framework

### 3.1. Organizational Classes and Probabilistic Representations

Section 2 introduced organization as the degree to which the identity-defining relationships among system states are preserved under transformation or perturbation. To operationalize this concept, a mathematical representation of organizational classes is required.

An organizational class consists of all admissible realizations that preserve the relationships necessary to maintain a recognizable identity. Membership in a class is therefore determined by the preservation of defining constraints rather than by exact agreement among individual states.

Figure 1 introduced the five-pointed star as a canonical organizational class. The same principles apply to physical systems such as polarized electromagnetic waves, whose identity is defined by polarization relationships rather than geometric constraints. The defining identity of the organizational class resides not in its energy, wavelength, or amplitude, but in the relationship among its polarization states.

**Figure 2:**
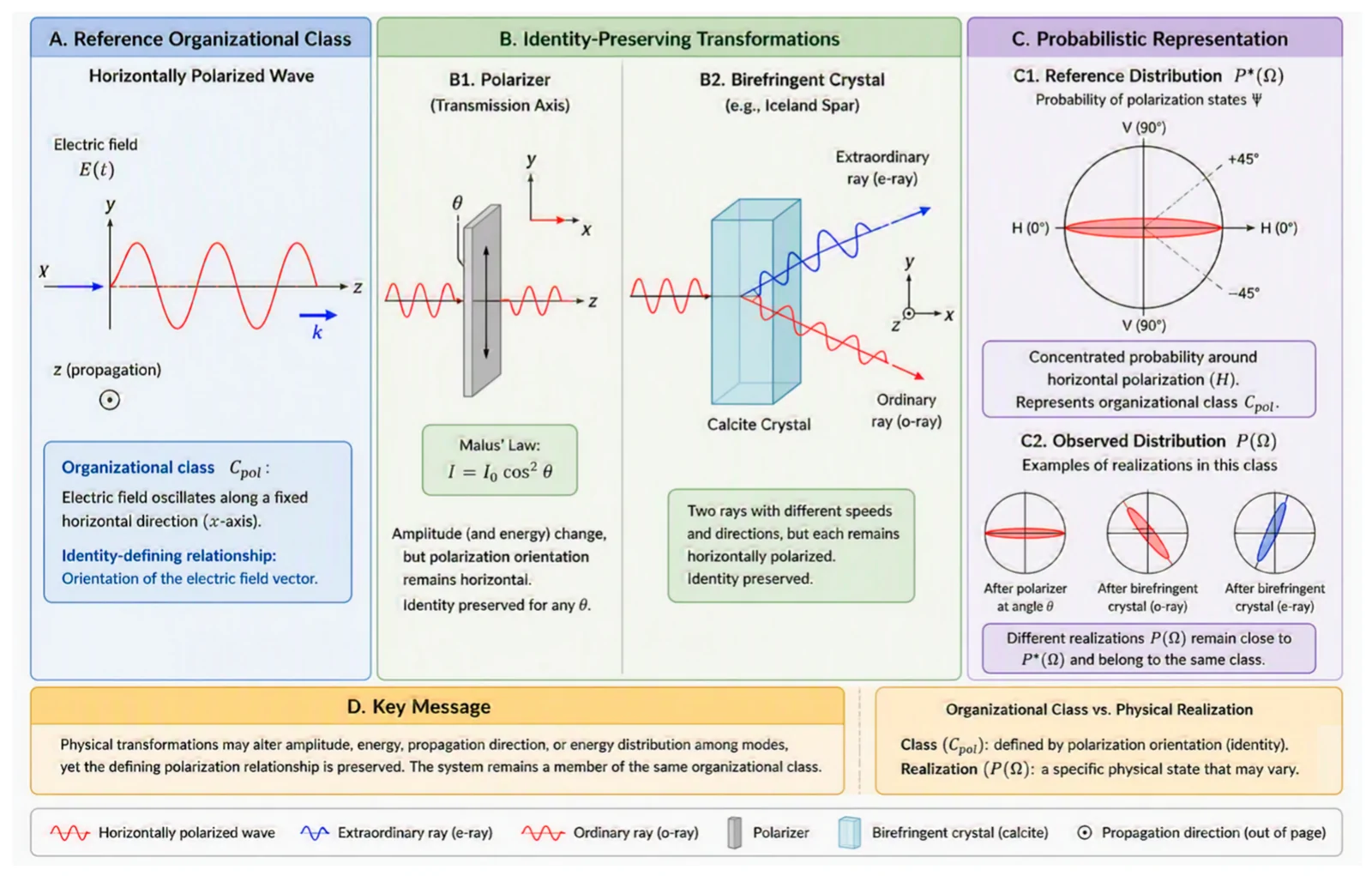
Physical realizations of an organizational class through polarization-preserving transformations. A linearly polarized electromagnetic wave defines the reference organizational class. Propagation through an aligned polarizer alters transmitted intensity while preserving polarization identity. Propagation through a birefringent crystal produces ordinary and extraordinary rays, modifying the physical realization of the wave while preserving the polarization relationships that characterize the class. These transformations demonstrate that substantial physical change may occur without necessarily altering organizational identity.

Figure 2 illustrates this concept using two common optical transformations. In the first case, a linearly polarized wave passes through a polarizing filter. Although the transmitted intensity may change, the defining polarization relationship remains preserved. In the second case, a birefringent crystal such as Iceland spar separates the incident wave into ordinary and extraordinary rays. The energy distribution and propagation paths change substantially, yet the polarization structure remains well defined and identifiable.

The examples of Figure 1 and Figure 2 illustrate two distinct manifestations of the same principle. In the geometric case, identity is defined by relationships among vertices and connecting segments. In the electromagnetic case, identity is defined by relationships among polarization states. Although these systems differ physically, both may be described as organizational classes characterized by identity-defining constraints.

The purpose of the mathematical framework developed below is to provide a common representation for such classes independent of their physical realization.

### 3.2. Generalized State Spaces

Let ω∈Ω denote an individual state within the generalized state space Ω. Probability distributions are therefore written as P(ω,t), while integrations are performed over the state space Ω. The elements of Ω may represent positions, frequencies, symbols, graph nodes, polarization states, molecular configurations, or any other domain-specific variables relevant to the system under consideration. The framework therefore remains independent of a particular physical implementation.

To accommodate a broad range of applications, a generalized state space may be represented as

Ω=(R,K,F,Ψ)

where

- R denotes spatial coordinates;
- K denotes momentum, propagation, or angular coordinates;
- F denotes spectral or frequency variables;
- Ψ denotes internal degrees of freedom such as phase, polarization, symbolic state, or other application-dependent descriptors.

For the polarization example of Figure 2, the organizational identity resides primarily within the Ψ component of the state space. For the five-pointed star of Figure 1, organizational identity is encoded primarily within the spatial relationships contained in R. Both systems therefore fit naturally within the same generalized representation despite their differing physical realizations.

A realization of a system is represented by a normalized probability distribution

P(Ω,t)

satisfying

$$\int_{\Omega }P(\Omega ,t)\;d\Omega =1.$$

The distribution describes the occupancy of states within the accessible state space and provides a probabilistic representation of a particular realization of an organizational class.

### 3.3. Entropic Occupancy and Organizational Structure

Entropy remains an important descriptor within the proposed framework.

For a distribution P(Ω,t), entropy is defined as

$$S=-k\int_{\Omega }P(\Omega ,t)ln\;P(\Omega ,t)d\Omega$$

where k is a proportionality constant.

Entropy characterizes the dispersion of state occupancies across the accessible state space. Maximum entropy occurs when occupancy becomes uniformly distributed,

$$P(\Omega )=\frac{1}{|\Omega |}.$$

Under this condition, no state is preferentially occupied and the distribution exhibits maximal statistical dispersion.

Within the present framework, entropy and organization are interpreted as complementary descriptors rather than competing quantities. Entropy characterizes the statistical distribution of state occupancies across an accessible state space, whereas organization characterizes the preservation of identity-defining relationships embedded within that occupancy. Both quantities may coexist simultaneously within the same system.

This distinction may be observed in the examples of Figure 1 and Figure 2. In Figure 1, the various realizations of the five-pointed star belong to the same organizational class despite differences in scale, orientation, texture, and graphical representation. Likewise, in Figure 2, polarization-preserving transformations alter aspects of the physical realization of the wave while preserving the defining relationships associated with the polarization class. In both cases, organizational identity remains preserved even though observable properties of the system have changed.

Organization should therefore not be interpreted as negative entropy, negentropy, entropy reduction, or the absence of disorder. Rather, entropy and organization describe different aspects of the same system. Entropy characterizes occupancy dispersion, while organization characterizes the preservation of identity-defining relationships among occupied states.

The ability of these quantities to evolve independently becomes particularly important when organizational degradation and recognition limits are considered. These concepts are introduced in subsequent sections.

### 3.4. Reference Organizational Models

An organizational class provides the conceptual definition of identity. To evaluate the preservation of that identity mathematically, a reference representation is required.

Let

$$P^{*}(\Omega )$$

denote the probabilistic representation of an organizational class. The distribution $P^{*}(\Omega )$ does not represent a single physical realization. Rather, it represents the defining occupancy structure associated with a recognizable class of systems.

This distinction is important. Individual realizations of an organizational class may differ substantially in appearance, energy distribution, scale, orientation, or physical implementation while preserving the same underlying identity. The role of $P^{*}\left(\Omega \right)$ is therefore not to describe a specific realization but to represent the organizational constraints that define class membership.

For the five-pointed star example introduced in Figure 1, $P^{*}\left(\Omega \right)$ represents the occupancy structure associated with the defining geometric relationships of the star class. Rotated, scaled, textured, or fractalized stars may correspond to different observed realizations while remaining associated with the same underlying organizational model.

Likewise, in the polarization example of Figure 2, $P^{*}\left(\Omega \right)$ represents the defining polarization structure of the organizational class. Propagation through a polarizer or birefringent crystal may alter the physical realization of the wave while preserving the underlying polarization identity represented by the reference model.

The construction of $P^{*}(\Omega )$ depends upon the domain of application. Reference organizational models may be derived from:

- Physical laws governing system behavior;
- Geometric or topological constraints;
- Communication protocols and coding structures;
- Functional requirements;
- Learned statistical models;
- Empirical observations of representative realizations.

Regardless of origin, the reference model defines the organizational class against which observed realizations are evaluated.

Importantly, $P^{*}\left(\Omega \right)$ should not be interpreted as an idealized or error-free state. In many systems, organizational classes may encompass a family of admissible realizations rather than a single configuration. The reference model therefore serves as a representation of organizational identity rather than a representation of perfection.

Within this framework, recognition becomes possible because observers, classifiers, or communication systems possess access, explicitly or implicitly, to such reference organizational models. Organizational evaluation can then be interpreted as the process of determining whether an observed realization remains sufficiently consistent with the defining constraints of its organizational class.

### 3.5. Organizational Realizations

The reference organizational model P*(Ω) introduced in the previous section represents the defining structure of an organizational class. Actual systems, however, are encountered as specific realizations of that class.

Let P(Ω) denote an observed realization of an organizational class represented by P*(Ω). The class establishes identity, whereas the realization provides a specific manifestation of that identity.

Multiple realizations may therefore belong to the same organizational class despite differences in appearance, energy distribution, scale, orientation, or physical implementation. Conversely, realizations exhibiting similar statistical properties may belong to different organizational classes when their identity-defining relationships differ.

The central problem becomes determining the extent to which an observed realization remains consistent with the organizational identity represented by the reference model. This motivates the introduction of organizational deviation.

### 3.6. Organizational Deviation

Organization is concerned with the preservation of identity-defining relationships. To quantify this preservation, it is necessary to estimate the deviation between an observed realization and its corresponding reference organizational model.

Let

$$P^{*}(\Omega )$$

denote the probabilistic representation of an organizational class and

P(Ω)

denote an observed realization.

We define the organizational deviation

$$\Delta_{O}$$

as a measure of departure between the observed realization and the reference class representation:

$$\Delta_{O}=D(P,P^{*})$$

where D(⋅) denotes an admissible measure of divergence between probability distributions.

Organizational deviation does not measure the loss of energy, the increase in uncertainty, or the dispersion of occupancy. Rather, it estimates the degree to which the observed realization departs from the defining occupancy structure associated with its organizational class. A small value of $\Delta_{O}$ indicates that the observed realization remains strongly consistent with the reference organizational model. Larger values indicate progressively greater departure from the defining structure of the class.

Importantly, the framework itself is independent of the specific divergence measure employed. Different organizational primitives may require different methods for comparing realizations and reference models. The choice of divergence measures therefore constitutes an implementation decision rather than a defining feature of the framework.

Several divergence measures may be appropriate depending on the application, including:

- Kullback–Leibler divergence for occupancy-based probabilistic comparisons [9];
- Jensen–Shannon divergence for symmetric probability comparisons [26];
- Wasserstein distance for geometry-aware comparisons involving spatial structure [27,28].

The choice among these measures depends upon the nature of the organizational constraints being represented. Different organizational classes may therefore require different operational measures of deviation.

For the remainder of this work, the Kullback–Leibler divergence is adopted because it naturally quantifies differences between probability distributions and is widely used within information theory and statistical signal processing [9,10]:

$$D_{KL}(P∥P^{*})=\int_{\Omega }P(\Omega )ln\left(\frac{P(\Omega )}{P^{*}(\Omega )}\right)d\Omega$$

where P(Ω) denotes an observed realization and $P^{*}(\Omega )$ denotes the corresponding reference organizational model.

Within the present formulation, $D_{KL}$ does not measure organization directly. Instead, it estimates the degree of organizational deviation between the observed realization and the organizational class represented by $P^{*}(\Omega )$.

As organizational deviation increases, the observed realization progressively departs from the defining constraints of its organizational class. Conversely, small divergence values indicate strong conformity with the identity-defining relationships represented by the reference model.

### 3.7. Recognition Boundaries

Organizational deviation provides a measure of departure between an observed realization and its corresponding reference organizational model. However, deviation alone does not determine whether organizational identity has been preserved.

Figure 1 and Figure 2 illustrate that substantial transformations may occur while recognizable identity remains intact. A rotated, scaled, or textured five-pointed star may continue to belong to the same organizational class despite observable differences from a reference realization. Likewise, a polarized electromagnetic wave may undergo attenuation, filtering, or birefringent splitting while preserving the defining polarization relationships associated with its organizational class. These examples demonstrate that organizational identity is generally tolerant to finite levels of variation.

When Kullback–Leibler divergence is employed, it is assumed that the support of the observed realization is contained within the support of the reference organizational model, $\mathrm{supp}(P)\subseteq \mathrm{supp}(P^{*})$, ensuring that the divergence remains finite. Alternative divergence measures may be preferred when this condition is not satisfied.

Recognition therefore plays a central role in determining whether a structure remains a member of a recognizable organizational class. An observed realization may differ from its reference model while still preserving sufficient identity-defining relationships for successful classification. Conversely, a realization may eventually depart so far from the reference structure that recognition is no longer possible.

**Figure 3:**
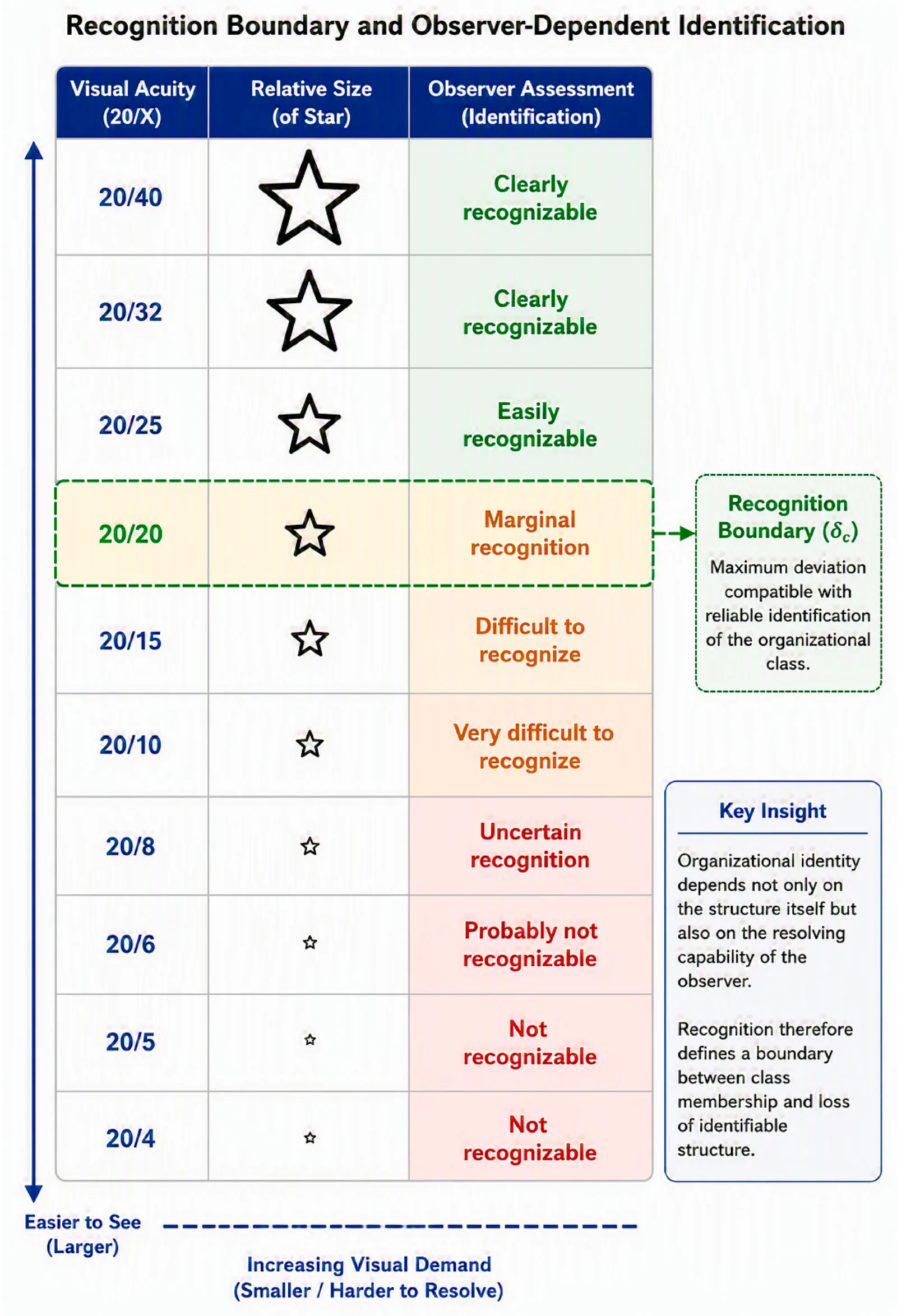
Recognition boundary and observer-dependent organizational identity. A five-pointed star is presented at progressively smaller relative sizes corresponding to increasing visual-acuity requirements. As the observable representation decreases relative to the resolving capability of the observer, recognition becomes progressively more uncertain. The highlighted threshold region illustrates the concept of a recognition boundary, δc separating realizations that remain reliably identifiable from those that no longer preserve recognizable identity. The figure demonstrates that organizational identity depends on both the observed structure and the capabilities of the observing system.

Figure 3 illustrates this concept using a simple visual-acuity example. A five-pointed star is presented at progressively smaller relative sizes. As the observable representation decreases relative to the resolving capability of the observer, identification becomes progressively more uncertain. The transition from reliable identification to failed identification illustrates the existence of a recognition boundary separating recognizable and unrecognizable realizations.

The visual-acuity example highlights an important property of organizational identity: recognition is inherently observer dependent. The same physical realization may be successfully identified by one observer while remaining unrecognizable to another. Recognition boundaries therefore depend not only on structural properties of the observed system but also on the capabilities, prior models, and decision criteria of the observing system.

This perspective is consistent with established theories of signal detection, classification, and pattern recognition, where recognition depends on both the observed signal and the characteristics of the observer or classifier [29,30,31].

To formalize this concept, we define the recognition boundary

$$\delta_{c}$$

as the maximum admissible organizational deviation compatible with reliable class membership.

The corresponding recognizable class may be expressed as

$$C=\left\{P(\Omega ):D(P,P^{*})\leq \delta_{c}\right\}$$

where

- $P^{*}(\Omega )$ represents the reference organizational model;
- P(Ω) represents an observed realization;
- $D(P,P^{*})$ represents the selected organizational deviation measure.

Realizations satisfying this condition preserve sufficient identity-defining relationships to remain members of the organizational class. Realizations exceeding the recognition boundary no longer satisfy the recognition criterion and are therefore considered organizationally distinct.

The recognition boundary serves as the critical bridge between organizational deviation and organizational preservation. It establishes the point at which differences between a realization and its reference organizational model cease to be interpreted as variation within a class and instead become evidence of membership in a different class or the loss of recognizable identity.

Recognition boundaries may be determined analytically, experimentally, operationally, or through classifier-performance criteria depending on the application domain.

### 3.8. Organization Measure

The recognition boundary introduced in the previous section establishes the maximum admissible organizational deviation compatible with reliable membership within an organizational class. Organizational deviation alone, however, does not provide a direct measure of the degree to which identity has been preserved. A normalized measure of organizational preservation is therefore required.

Let

$$\Delta_{O}=D(P,P^{*})$$

denote the organizational deviation between an observed realization P(Ω) and its corresponding reference organizational model $P^{*}(\Omega )$.

Within the present probabilistic realization, organization is estimated by

$$O(P)=max\left(0,1-\frac{\Delta_{O}}{\delta_{c}}\right)$$

where

- O(P) denotes the organization of the observed realization;
- $\Delta_{O}$ denotes the organizational deviation;
- $\delta_{c}$ denotes the recognition boundary associated with the organizational class.

Under this formulation:

O(P)=1

corresponds to complete conformity with the reference organizational model,

0<O(P)<1

corresponds to partially degraded but recognizable realizations, and

O(P)=0

corresponds to realizations that exceed the recognition boundary and no longer preserve recognizable organizational identity.

This interpretation reflects the conceptual framework introduced in Section 2. Organization is not a direct measure of occupancy, energy, uncertainty, or information content. Rather, it quantifies the degree to which identity-defining relationships remain preserved relative to the constraints that define an organizational class.

Importantly, organization is defined relative to a reference model and a recognition boundary. Consequently, organizational values may depend upon the characteristics of the observing system, the selected representation, and the particular organizational class under consideration.

### 3.9. Organization–Deorganization Ratio

While organization provides a normalized measure of structural preservation, it is often useful to characterize the balance between organizational integrity and organizational deviation.

We therefore define the Organization–Deorganization Ratio (ODR) as

$$ODR=\frac{O(P)}{\Delta_{O}}$$

for $\Delta_{O}>0$.

The ODR provides a measure of the relative dominance of organizational preservation over organizational degradation.

When:

$$\Delta_{O}\ll \delta_{c}$$

organization dominates and

ODR≫1.

As organizational deviation increases and approaches the recognition boundary,

$$\Delta_{O}\to \delta_{c},$$

the ODR decreases toward zero. For $\Delta_{O}=0$, the ODR is interpreted as the limiting case of complete organizational conformity. The ODR may therefore be interpreted as a structural analogue to traditional signal-to-noise ratio concepts. Rather than comparing energy contributions, however, the ODR compares organizational preservation to organizational degradation.

This interpretation is particularly useful in communication, sensing, and recognition systems where successful operation depends less on absolute signal energy than on the ability to preserve recognizable organizational structure.

### 3.10. Operational Interpretation

The mathematical framework developed in this section provides a common procedure for evaluating organizational preservation across a broad range of physical, informational, biological, and symbolic systems.

The process may be summarized as follows:

1. Define an organizational class through its identity-defining constraints.
2. Represent the organizational class using a reference organizational model $P^{*}(\Omega )$.
3. Represent the observed realization using P(Ω).
4. Estimate organizational deviation:
  $$\Delta_{O}=D(P,P^{*}).$$
5. Determine the recognition boundary:
  $$\delta_{c}$$
  for the relevant observer, classifier, or receiver.
6. Evaluate organization:
  O(P)
  and the corresponding organization–deorganization ratio.

Within this framework, entropy and organization coexist as complementary descriptors of system behavior. Entropy characterizes occupancy dispersion within an accessible state space, whereas organization characterizes the preservation of identity-defining relationships embedded within that occupancy. Organizational deviation and recognition boundaries provide the mechanisms through which the persistence of recognizable structure may be evaluated quantitatively.

The following section illustrates the application of these concepts through a series of geometric, perceptual, and communication-based examples that demonstrate the relationship between entropy, organization, recognition, and observability.

## 4. Illustrative Applications

The organizational framework developed in Section 2 and Section 3 provides a general method for representing organizational classes, quantifying organizational deviation, and evaluating the preservation of recognizable identity. The following examples illustrate how the framework may be applied to structured systems undergoing perturbation, observation, and transmission.

The examples are intentionally simple and are intended to clarify the interpretation of organizational classes, recognition boundaries, and organizational preservation. Although the examples employ geometric structures and communication analogies, the underlying framework is applicable to any system in which recognizable identity emerges from identity-defining relationships among states.

### 4.1. Organization Degradation in a Maximum-Entropy Background

A useful illustration of the distinction between entropy and organization is provided by a structured object embedded within a statistically uniform background.

Consider a discrete spatial system defined over a finite grid,

Ω={(i,j)},

where each location may occupy one of two possible states. A maximum-entropy background is constructed by assigning equal probability to each state,

$$P_{bg}(x_{ij}=1)=0.5.$$

The resulting field appears as a statistically homogeneous gray background with maximal occupancy uncertainty.

Within this background, a realization belonging to the five-pointed-star organizational class is introduced by imposing relationships among a subset of states. The resulting structure represents a foreground organizational realization embedded within a maximum-entropy occupancy field.

Progressive perturbation is then applied only to the organizational realization while leaving the background occupancy statistics unchanged. As perturbation increases, the defining relationships associated with the star class are gradually disrupted.

**Figure 4:**
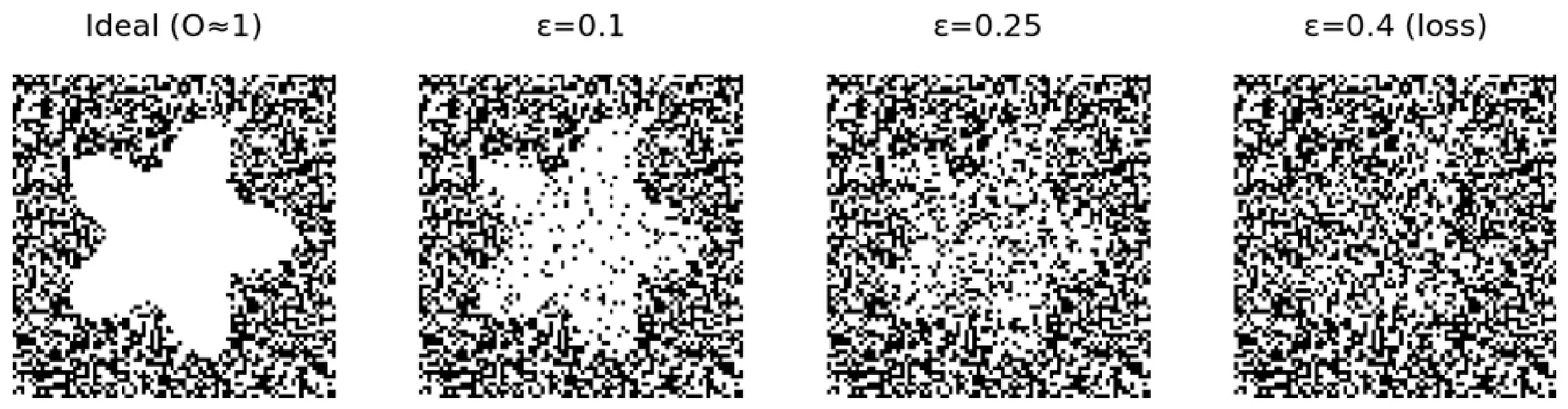
Organization degradation in a maximum-entropy background. A realization belonging to the five-pointed-star organizational class is embedded within a binary maximum-entropy field. Increasing perturbation progressively disrupts the identity-defining relationships associated with the organizational class while leaving the background occupancy statistics largely unchanged. The foreground structure becomes progressively less recognizable until recognizable identity is eventually lost. The example illustrates that organization and entropy characterize different aspects of the same system.

The resulting degradation sequence is shown in Figure 4.

As organizational deviation increases, the observed realization progressively departs from the reference organizational model. The visible structure becomes increasingly difficult to recognize even though the global occupancy statistics of the field remain nearly unchanged.

This example illustrates one of the central ideas of the present work: Entropy characterizes the statistical distribution of occupancy across the state space, whereas organization characterizes the preservation of recognizable foreground structure embedded within that occupancy.

Consequently, organizational degradation may occur while entropy remains approximately constant. The framework therefore permits systems to exhibit substantial changes in organization without requiring correspondingly large changes in occupancy dispersion.

### 4.2. Recognition and Observer Dependence

The concept of organizational preservation is inherently linked to recognition. As discussed in Section 3.8, recognition depends not only on the structure being observed but also on the capabilities of the observing system. Figure 3 illustrated this principle through the progressive reduction in a recognizable symbol relative to the observer’s resolving capability.

A realization may therefore remain physically unchanged while yielding different recognition outcomes for different observers. Changes in visual acuity, detector sensitivity, classifier performance, or measurement resolution may alter the effective recognition boundary associated with an organizational class.

A familiar example is provided by optical imaging. A structure that is readily identifiable at high resolution may become ambiguous or unrecognizable when observed at lower resolution. The underlying physical structure may remain intact, yet the observable realization no longer preserves sufficient information to satisfy the recognition criteria.

This observation highlights an important property of organization: Organizational identity is not determined solely by the observed structure but also by the ability of a receiver to resolve and interpret the identity-defining relationships associated with that structure.

Recognition boundaries are therefore operational quantities whose values may depend on the observer, measurement system, or classifier used to evaluate organizational class membership.

### 4.3. Organization Under Channel Constraints and Observability

**Figure 5:**
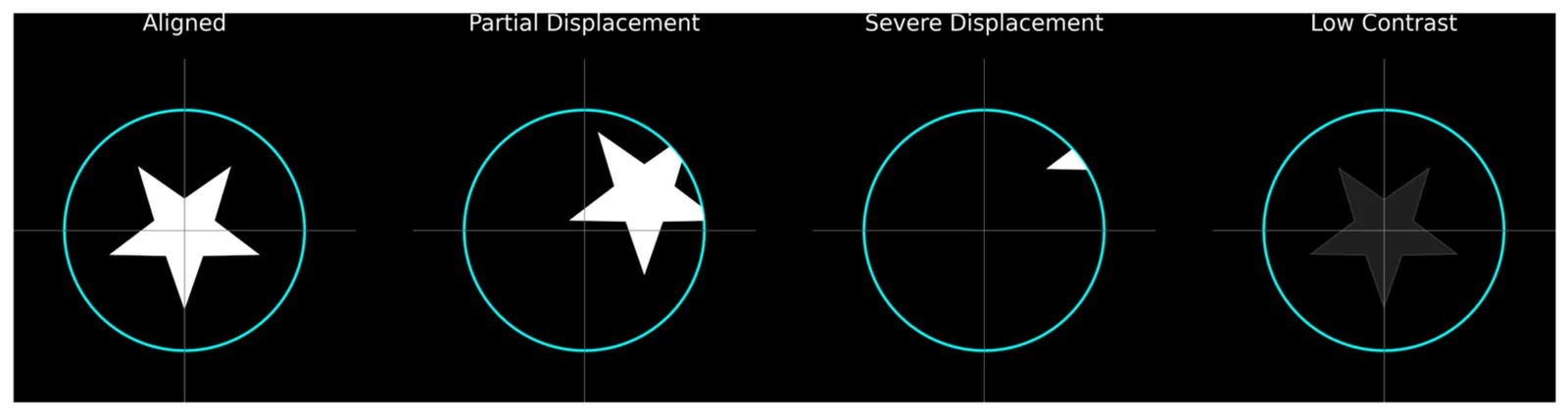
Organization under channel constraints and observability. A structured realization is observed through a restricted observable region corresponding to a communication channel. As the observable portion of the realization decreases, organizational deviation increases and recognizable identity becomes progressively more difficult to preserve. The final panel illustrates a distinct mechanism in which reduced contrast limits observability despite the physical preservation of the structure.

Organizational preservation may also be affected by limitations imposed by communication channels and observation systems. Consider the geometric analogy illustrated in Figure 5, where a realization belonging to an organizational class is observed through a restricted aperture representing a communication channel.

When the organizational realization remains fully observable, the observed distribution remains strongly consistent with the reference organizational model and recognizable identity is preserved.

As the observable region becomes restricted, only a fraction of the identity-defining relationships remains accessible to the observer. Organizational deviation therefore increases even though the underlying structure itself remains unchanged.

A second mechanism arises from limitations in observability. A structure may remain fully present within the observable region yet fail to be recognized because insufficient contrast, resolution, or detector sensitivity prevents reliable discrimination from the background.

These examples demonstrate that communication channels do more than transport energy or information; they constrain the portion of organizational structure that remains observable to a receiver. Successful communication therefore depends on preserving sufficient identity-defining relationships for recognition to remain possible.

While Figure 4 and Figure 5 illustrate these concepts qualitatively, the same principles may be examined quantitatively by representing an organizational class as a probability distribution and measuring its deviation from a reference model. The following example develops this idea using a Gaussian organizational class observed through a finite channel, providing an explicit calculation of organizational deviation, recognition boundaries, and organizational preservation within the proposed framework.

### 4.4. Numerical Illustration Using a Gaussian Organizational Class

To provide a simple quantitative demonstration of the proposed framework, consider an organizational class represented by a Gaussian probability distribution

$$P^{*}(x)=\frac{1}{\sqrt{2\pi \sigma^{2}}}exp\left(-\frac{x^{2}}{2\sigma^{2}}\right),$$

with mean zero and variance $\sigma^{2}$.

Assume that the organizational class is communicated through a channel whose observable range is limited to

−2σ≤x≤+2σ.

For a Gaussian distribution, approximately 95.45% of the total probability mass lies within this interval. We therefore adopt

$$\delta_{c}=0.9545$$

For illustrative purposes, the recognition boundary is chosen to coincide with the observable channel extent. In general, recognition boundaries need not correspond directly to a fixed probability mass. Realizations that remain sufficiently concentrated within this range are considered recognizable members of the organizational class.

To model organizational degradation, we consider a family of observed realizations

$$P(x)=N(\mu ,\sigma^{2}),$$

in which the variance remains unchanged while the mean is progressively displaced relative to the reference organizational model. For Gaussian distributions having identical variances, the Kullback–Leibler divergence is given by

$$D_{KL}(P∥P^{*})=\frac{\mu^{2}}{2\sigma^{2}}.$$

For simplicity, we set

σ=1.

The organization measure introduced in Section 3 is then

$$O(P)=max\left(0,1-\frac{D_{KL}(P∥P^{*})}{\delta_{c}}\right).$$

Four representative cases are examined.

#### 4.4.1. Case 1: Perfect Alignment


μ=0

The observed realization coincides with the reference organizational model.

$$D_{KL}=0$$

O(P)=1.

The realization exhibits complete organizational conformity.

#### 4.4.2. Case 2: One Standard Deviation Displacement


μ=1

The divergence becomes

$$D_{KL}=\frac{1^{2}}{2}=0.50.$$

The corresponding organization is

$$O(P)=1-\frac{0.50}{0.9545}=0.476.$$

The realization remains recognizable but exhibits substantial organizational degradation.

#### 4.4.3. Case 3: Two Standard Deviation Displacement


μ=2

The divergence becomes

$$D_{KL}=\frac{2^{2}}{2}=2.00.$$

Since

$$D_{KL}>\delta_{c},$$

the realization lies beyond the recognition boundary.

O(P)=0.

Recognizable organizational identity is no longer preserved.

#### 4.4.4. Case 4: Three Standard Deviation Displacement


μ=3

The divergence becomes

$$D_{KL}=\frac{3^{2}}{2}=4.50.$$

Again,

$$D_{KL}>\delta_{c},$$

and therefore

O(P)=0.

The realization is organizationally distinct from the reference class.

As shown in Table 1, this example illustrates the complete organizational framework in a simple analytical setting. A reference organizational class is represented by $P^{*}(x)$, an observed realization is represented by P(x), organizational deviation is quantified through $D_{KL}(P∥P^{*})$, and recognizable identity is determined relative to a recognition boundary $\delta_{c}$. As displacement increases, organizational deviation grows and organization decreases until the realization can no longer be considered a recognizable member of the original organizational class. Unlike the preceding geometric examples, this example provides a fully quantitative demonstration of how organizational preservation may be evaluated within the proposed probabilistic framework.

## 5. Discussion

### 5.1. Organization and Structural Identity

The central premise of this work is that many systems derive their recognizable identity not from individual states alone, but from the relationships that connect those states. Traditional descriptors such as energy, entropy, and information characterize important statistical and physical properties of a system, yet they do not explicitly address whether the defining relationships necessary for recognition remain preserved.

To address this limitation, organization was introduced as a descriptor of structural identity. Within the proposed framework, organization is defined as the degree to which identity-defining relationships among system states are preserved under transformation or perturbation. This interpretation shifts attention from individual states to the constraints that define membership within an organizational class.

The examples developed throughout this work demonstrate that substantial changes in physical realization may occur without destroying recognizable identity. Organization therefore captures an aspect of system behavior that is complementary to traditional statistical and information-theoretic descriptors.

### 5.2. Organization and Entropy

A principal result of this work is the explicit distinction between entropy and organization. Entropy characterizes the statistical dispersion of occupancy across an accessible state space, whereas organization characterizes the preservation of recognizable structure embedded within that occupancy. The two quantities therefore describe different aspects of the same system.

This distinction is illustrated most clearly by the degradation example of Figure 4, where a realization belonging to the five-pointed-star organizational class progressively loses recognizable structure while the statistical properties of the surrounding background remain largely unchanged. In this case, organization decreases substantially while entropy changes only minimally.

A useful interpretation is to view entropy as a descriptor of occupancy and organization as a descriptor of structural identity. Under this perspective, organization should not be interpreted as negentropy, entropy reduction, or the inverse of disorder. Rather, organization complements entropy by describing whether recognizable structure remains preserved within a system.

The proposed framework may be situated relative to established descriptors commonly used in physics, information theory, and communication systems (Table 2).

Each of these quantities characterizes a distinct aspect of system behavior. Energy and entropy describe physical and statistical properties of state occupancy, while divergence and mutual information quantify relationships between distributions or variables. Organization complements these quantities by explicitly addressing the persistence of recognizable identity.

### 5.3. Recognition, Observability, and Communication

The framework developed here places recognition at the center of organizational preservation. Recognition therefore acts as the operational criterion linking organizational deviation to organizational preservation.

This perspective has important implications for communication and sensing systems. The examples of visual acuity, channel constraints, and observability demonstrate that organizational preservation depends not only on the transmitted structure but also on the ability of a receiver to resolve and interpret that structure.

From this viewpoint, communication systems may be understood as organization-preservation systems. The role of a channel is not merely to transport energy or information, but to preserve enough organizational structure for recognizable identity to survive transmission. Likewise, sensing systems are constrained not only by physical signal levels but also by the ability to observe and reconstruct the relationships necessary for recognition.

## 6. Conclusions

This work introduced a framework in which organization is interpreted as the preservation of identity-defining relationships among system states under transformation or perturbation. Unlike traditional descriptors that characterize magnitude, uncertainty, or statistical dependence, the proposed framework focuses explicitly on the persistence of recognizable structure.

The theoretical development began by introducing the concepts of identity, organizational classes, and recognition. These concepts were then formalized through reference organizational models, organizational deviation measures, recognition boundaries, and a normalized estimate of organizational preservation. Within this framework, organization is not defined by a particular divergence measure but by the persistence of identity-defining relationships, with probabilistic representations providing one operational implementation.

A central result of this work is the explicit distinction between entropy and organization. Entropy characterizes the statistical distribution of state occupancies, whereas organization characterizes the preservation of recognizable structural identity embedded within that occupancy. The illustrative examples demonstrate that organization and entropy may coexist and evolve independently, allowing substantial organizational degradation to occur while global occupancy statistics remain largely unchanged.

The principal contributions of this work may be summarized as follows:

- Organization is defined as the preservation of identity-defining relationships within an organizational class.
- Organizational classes are introduced as collections of realizations sharing a common recognizable identity.
- Recognition boundaries provide an operational criterion for determining whether organizational identity is preserved.
- Entropy and organization are shown to represent complementary rather than competing system descriptors.
- Communication and sensing systems are interpreted as processes that preserve, transform, or degrade organizational structure.
- Observability is identified as a critical factor influencing organizational preservation and recognition.

The framework presented here is an initial step toward a broader theory of organization. Future work will focus on alternative representations of organizational classes, experimental determination of recognition boundaries, applications to high-dimensional systems, and the role of organization in communication, machine learning, biological systems, and complex adaptive systems.

Taken together, these results suggest that organization provides a useful complementary descriptor for systems in which recognizable identity plays a central role. By combining entropy-based descriptions of occupancy with organization-based descriptions of structural preservation, the proposed framework offers a foundation for studying how organized structures emerge, persist, transform, and degrade across diverse domains.

## Figures and Tables

**Table 1:** Organizational degradation of a Gaussian organizational class under progressive displacement.

Mean Shift (μ/σ)	$D_{KL}(P∥P^{*})$	Organization O(P)	Interpretation
0	0.00	1.000	Complete conformity
1	0.50	0.476	Recognizable but degraded
2	2.00	0.000	Beyond recognition boundary
3	4.50	0.000	Organizational identity lost

**Table 2:** Relationship between organization and established system descriptors.

Descriptor	Primary Role
Energy	Magnitude of activity
Entropy	Occupancy dispersion
Divergence Measures	Deviation between realizations and reference models
Mutual Information	Statistical dependency
Organization	Preservation of identity-defining relationships

## Data Availability

No new data were created or analyzed in this study. Data sharing is not applicable to this article.

## Abbreviations

The following abbreviations are used in this manuscript:

SNR	Signal-to-Noise Ratio
ODR	Organization–Deorganization Ratio
KL	Kullback–Leibler
PDF	Probability Distribution Function
